# Resilience in rural left-behind middle school students in Yunyang county of the Three Gorges area in China: a prospective cohort study

**DOI:** 10.1186/s12888-016-0781-1

**Published:** 2016-03-23

**Authors:** Yan Luo, Hong Wang, Xun Lei, Xue Guo, Ke Huang, Qin Liu

**Affiliations:** School of Public Health and Management, Research Center for Medicine and Social Development, Innovation Center for Social Risk Governance in Health, Chongqing Medical University, Chongqing, 400016 China; Chongqing Traditional Chinese Medicine Hospital, Chongqing, 400021 China; Public Health Clinical Center of Chengdu, Sichuan, 610066 China

**Keywords:** Resilience, Left-behind children, Middle school students

## Abstract

**Background:**

The number of left-behind children in China is gradually increasing. This study aimed to understand the mental health status and changes in resilience of rural, left-behind middle school students in Yunyang County of the Three Gorges area in China.

**Methods:**

A prospective cohort study, including two follow-up surveys, with a frequency of once every 6 months was conducted among middle school students in Yunyang County. A self-designed questionnaire was used to obtain socio-demographic factors of participants, the Mental Health Test (MHT) scale was used to assess their mental health statuses, and resilience levels were collected using Resilience Youth Development Module (RYDM) scale at baseline and at the first and second follow-up investigations.

**Results:**

Of the 1401 students who completed the baseline survey, 1322 students were eligible for the cohort study, of whom 1160 were investigated in the first follow-up survey. Ultimately, 1101 students completed the 1-year cohort. The detection rate of mental health problems for middle school students in the rural Three Gorges area was 5.64 %, and there was no significant difference between left-behind students (LBS) and non-left-behind students (NLBS) (*χ*^2^ = 1.056, *P* = 0.304). The detection rates of medium resilience rose gradually (Z = 4.185, *P* = 0.000), while that of high resilience declined gradually (Z =−4.192, *P* = 0.000) in the baseline, first and second follow-up investigations. There was no significant difference between LBS and NLBS in resilience level (*P* > 0.05). The average RYDM scores were 2.990, 2.926, 2.904 among LBS in the baseline, first and second follow-up investigation, respectively, and the effect of time on the average RYDM scores was significant (F = 14.873, *P* = 0.000). The average MHT scores in LBS were 41.54, 39.79, 38.84 in the baseline, first and second follow-up investigations, respectively, and the detection rates of students who had psychological problems increased gradually (Z = 4.651, *P* = 0.000). The simple correlation coefficients between the RYDM and MHT scores were−0.227,−0.158, and−0.204 in the baseline, first and second follow-up survey, respectively (*P* = 0.000).

**Conclusions:**

The detection rate of mental health problems for middle school students in the rural Three Gorges area is relatively low and most of the LBS in area have medium or high resilience. The mental health status of LBS positively correlated with their resilience levels. Resilience declined gradually as time went on, but further studies with longer follow-up durations are needed to confirm the variation of resilience.

## Background

China has been undergoing rapid urbanization in the last few decades. By 2013, over 166 million people in China were internal migrants [1], forced to move from rural to urban areas to seek employment opportunities while their children stayed at home; hence the term “left-behind children (LBC)” [2, 3]. According to the 6th national population census, the number of LBC in rural China reached 61 million, accounting for 37.7 and 21.88 % of rural children and all children in China, respectively, and the total number of LBC in the Midwestern areas of China exceeded 50 % of all LBC in China [4]. There were about 900,000 LBC in the rural Three Gorges area, accounting for 38 % of the total number of LBC in Chongqing, China [5]. Our study focused on middle school LBC, who were specifically defined as “middle school students who lived and studied at their original rural residence while one or both parents migrated to a city for work for at least 6 months.” Middle school students in this study referred to both junior and senior high school students. These students were undergoing puberty, with rapid physical and psychological development. They grew up in incomplete families, and the lack of parental care and guidance may have caused them to encounter various mental health problems [6, 7], behavioral issues [8, 9] and other illnesses [10] more easily.

In recent years, studies about the mental health of left-behind students (LBS) have gradually increased, and most of these studies were focused on the risk factors associated with mental health [7, 11]. Not all adolescents with risk factors have mental health problems; thus, resilience as a measurement is concerned with mental health from a perspective of positive psychology [12–15]. There has been no uniform definition for resilience. An accepted operational definition of resilience is the state of being able to cope well with challenges after having encountered adversity [16]. Previous studies mostly focused on the theory, mechanism and influential factors of resilience [17–20], and most of them were cross-sectional studies, mainly used convenience sampling and the sample sizes were relatively small. Until now, there has been no cohort study about resilience among rural LBS in the Three Gorges area of China; therefore, our study aimed to investigate the resilience and mental health status of rural left-behind and non-left-behind middle school students in Yunyang County of the Three Gorges area of China, and to observe the change in resilience and mental health of these students.

## Methods

### Study settings

The Three Gorges area, as one of the most economically underdeveloped regions in China, has extreme geographic features and low agricultural and industrial productivity levels. Most farmers are forced to move away from their home to seek employment opportunities, leaving their children at home. Our study was conducted in Yunyang County, Chongqing, China, which is located in the center of the rural Three Gorges area and is one of the first immigrant counties with more than 100,000 LBC.

### Participants and sampling

Firstly, according to a multi-staged stratified cluster random sampling method, Yunyang County was chosen from the rural Three Gorges area. Second, considering the geographic location, economic conditions and demographic characteristics, three townships of Yunyang County and one middle school from each selected township were selected as the sampling areas. Then, three to five classes were randomly selected from the junior grade and from the senior grade in each middle school. A total of 23 classes with 1401 students were selected in the baseline survey. The study was approved by the Medical Ethics Review Committee of Chongqing Medical University. All subjects completed informed consent. Informed consents by both the students and their guardians were sought by signing informed consent forms.

### Baseline survey

During October 2012, the baseline survey was conducted in whole, selected classes with the assistance of school leaders and teachers. Before the survey, we illustrated the objectives and main contents of the study to the teachers, students, and guardians. Students were asked to complete the self-administered questionnaires under the guidance of the investigators, including the self-designed questionnaire, Resilience Youth Development Module (RYDM) scale and Mental Health Test (MHT) scale.

A self-designed questionnaire was used to obtain the socio-demographic factors including age, gender, living situation (in school or out of school), siblings (yes or no), marital status of parents (divorced or not divorced), subjective economic status (five levels from ‘very good’ to ‘very bad’), type of guardians (mother, father, grandparents, other relatives or friends), conflict with the guardian (including ‘no contradiction’, ‘contradicting sometimes’, or ‘contradicting frequently’), students’ worry about their parents working outside of the county (five levels from ‘not at all’ to ‘extremely worried’) and working status of parents (whether the student’s father, mother, or both parents worked outside the county).

The RYDM scale [21] was a part of the California Healthy Kids Survey (CHKS) designed by the Safe and Healthy Kids Program Office (SHKPO), which provided comprehensive and balanced coverage of both external (twelve dimensions) and internal (six dimensions) resilience assets. The scale consisted of 56 items, 51 of which concerned external and internal factors (four answer choices ranging from “Not at All True” to “Very Much True”, scoring 1, 2, 3, or 4, respectively), and the remaining five items concerned additional school factors (five answer choices ranging from “Strongly Disagree” to “Strongly Agree”, scoring 1, 2, 3, 4, or 5, respectively). Resilience was classified into low levels (scoring below 2), medium levels (scoring 2 to 3) and high levels (scoring above 3) according to the average scores of the students. Translated and revised versions in Chinese were used in our study [22].

The MHT scale [23] was revised according to the “diagnostic test of anxiety tendency” designed by Kurt Suzuki, which was widely used to assess the mental health of middle school students. The scale contains 100 items including one validity subscale and eight content subscales, and each item was scored 1 for “yes” and 0 for “no”, respectively. Mental health would be assessed by the total score of anxiety tendency, which means the higher of the score, the higher degree of anxiety, and the worse the children’s mental health status. Students who achieved a total score of 65 or higher had psychological problems.

Reliability and validity of the RYDM and MHT scales were psychometrically qualified in the children of the rural Three Gorges area. The Cronbach’s alpha coefficient, Spearman-Brown coefficient and test-retest reliability of the RYDM scale in our study were 0.945, 0.865, 0.838, respectively, and the correlation coefficients between total scale and sub-scales of the RYDM scale were 0.896 and 0.974, respectively, which were in accordance with a previous study [24]. The Cronbach’s alpha coefficient, Spearman-Brown coefficient and test-retest reliability of the MHT scale in our study were 0.897, 0.862, 0.976, respectively and the correlation coefficients between total scale and sub-scales ranged from 0.521 to 0.783, which were also in accordance with previous studies focused on LBC [25, 26].

### Cohort study

Of the 1401 students who completed the baseline survey, we excluded those considered to have mental illness or mental health problems based on the MHT scales (*n* = 79). The remaining 1322 students, including 986 LBS and 336 NLBS, were chosen to complete a 1 year follow-up observation; thus, a total of two follow-ups with a frequency of one every 6 months were conducted. All questionnaires and survey methods were the same as the baseline survey. 1160 students (860 LBS and 300 NBLS) and 1101 students (822 LBS and 279 NLBS) were investigated in the first and second follow-up surveys, respectively. The loss rate was 12.25 and 16.72 %, respectively, and there were no significant differences in the loss rates between LBS and NLBS in the first (*χ*^2^ = 0.933, *P* = 0.319) and second (*χ*^2^ = 0.020, *P* = 0.888) follow-up surveys. Of the 221 students lost in the follow-up surveys, 168 students transferred to another school, 47 dropped out of school and six students were on sick leave. Significant differences between LBS and NLBS were found in the comparison of siblings, living situation and economic status in the baseline survey (*P* < 0.05). Significant differences were also found in the comparison of grade, living situation and economic status in LBS and NLBS between baseline and the last follow-up survey (*P* < 0.05). The socio-demographic factors are shown in Table 1.

**Table 1:** The changes in the socio-demographic factors of participants between the baseline and the last follow-up survey

Basic information	Classfication	LBS				NLBS			
		The baseline survey (986)	The last follow-up survey (822)	*χ* ^2^	*P*	The baseline survey (336)	The last follow-up survey (279)	*χ* ^2^	*P*
Gender	Male	585	459	2.239	0.135	195	176	1.622	0.203
	Female	401	363			141	103		
Grade	Junior grade one	488	451	5.185	0.023	159	144	1.123	0.289
	Senior grade one	498	371			177	135		
Siblings	Yes	884	750	1.296	0.255	283	237	0.061	0.806
	No	102	72			53	42		
Living situation	In school	664	506	6.571	0.010	198	156	0.567	0.451
	Out of school	322	316			138	123		
Parents’ marital status	Divorced	71	61	0.032	0.858	17	18	0.55	0.458
	Not divorced	915	761			319	261		
Economic status	Good	41	21	24.822	0.000	33	11	10.427	0.005
	Medium	720	681			240	226		
	Bad	225	120			63	42		

*Abbreviations*: *LBS* left-behind students, *NLBS* non-left-behind students

### Statistical analysis

The completed questionnaires were coded, and then double entered into computer and cross checked by two researchers. Epidata 3.0 software (Jens M. Lauritsen, Michael Bruus and Mark Myatt, Odense, Denmark) was used to build our database and data management system. The data were analyzed using SPSS 17.0 (Chicago, SPSS Inc., IL, USA) and SAS 9.2 (SAS Institute, Cary, NC, USA). We calculated the detection rates of mental health problems and resilience levels among middle school students. Chi-square tests were used to analyze the comparison of socio-demographic factors, loss rate, detection rate of mental health problems and resilience levels between LBS and NLBS. The chi-square test for trend was used to analyze the trend of resilience levels and the LBS who have psychological problems. Repeated measure analysis of variance was used to compare the average RYDM scores in two groups. Simple correlation and linear regression analysis were used to analyze the association between the scores and the changes of RYDM and MHT scores. The *P* value of 0.05 for any test was considered to be statistically significant.

## Results

### The demographic characteristics of participants

Ultimately, 1101 students aged 9 to 18 (M = 14) completed our survey, including 822 LBS and 279 NLBS, 634 boys and 467 girls, 594 junior high school students and 507 senior high school students. There were no significant differences between LBS and NLBS by grade, living situation, marital status of parents and the subjective economic conditions (*P > 0.05*), while more students in the LBS group were boys and had siblings (*P < 0.05*).

### Detection rate of mental health problems and resilience levels change over time

The detection rate of mental health problems among middle school students in the rural Three Gorges area was 5.64 % (*n* = 1401), and there was no significant difference between LBS and NLBS (6.01 % vs 4.55 %, *χ*^2^ = 1.056, *P* = 0.304). After excluding those considered to have mental illness or mental health problems based on the MHT scale, the detection rate of LBS who had mental health problems were 0.00, 2.71 and 4.75 % in the baseline, first and second follow-up surveys, respectively (Z = 4.651, *P* = 0.000).

The detection rate of low, medium and high resilience among middle school students in the rural Three Gorges area was 1.00, 53.32, 45.68 %, respectively (*n* = 1401) and were 0.95, 54.15 and 44.90 %, respectively, for LBS. After excluding those with mental illness or those who had mental health problems based on the MHT scale, the detection rates of low resilience in LBS were 0.86, 1.48, 0.85 %, medium resilience were 50.86, 57.44, 61.14 %, and those of high resilience were 48.27, 41.08, 38.00 % in the baseline, first and second follow-up investigations, respectively. The detection rates of medium resilience rose gradually (Z = 4.185, *P* = 0.000) while that of high resilience declined (Z =−4.192, *P* = 0.000). There was no significant difference between LBS and NLBS in resilience level (*P* > 0.05). The changes of resilience are shown in Table 2.

**Table 2:** The changes in resilience of participants over time (*n* = 1101)

Time of investigation	Number	Low resilience (average score < 2)	Medium resilience (2 ≤ average score ≤ 3)	High resilience (average score >3)	*χ* ^2^	*P*
Baseline survey	LBS(810)	7 (0.86 %)	412 (50.86 %)	391 (48.27 %)	0.534	0.767
	NLB(291)	3 (1.03 %)	141 (48.45 %)	147 (50.52 %)		
The first follow-up survey	LBS(813)	12 (1.48 %)	467 (57.44 %)	334 (41.08 %)	2.075	0.354
	NLB(288)	8 (2.78 %)	161 (55.90 %)	119 (41.32 %)		
The second follow-up survey	LBS(821)	7 (0.85 %)	502 (61.14 %)	312 (38.00 %)	1.433	0.478
	NLB(280)	3 (1.07 %)	160 (57.14 %)	117 (41.79 %)		

*Abbreviations*: *LBS* left-behind students, *NLBS* non-left-behind students

### Changes in average RYDM scores over time

The average RYDM scores were 2.990, 2.926, 2.904 in LBS and 2.996, 2.895, 2.920 in NLBS at the baseline, first and second follow-up investigations, respectively. Both LBS and NLBS scored in the medium resilience range (scoring 2 to 3), and the average scores in LBS declined continuously.

Mauchly’s test of sphericity showed that the repeated measurement data were correlated (*χ*^2^ *=* 26.470*, P* = 0.000). Repeated measures analysis of variance showed that the effect of time on the average RYDM scores was significant (F = 14.873, *P* = 0.000, Table 3), which means the average RYDM scores were different in at least two survey times. However, there was no significant difference in the interaction effect of time and groups on the average RYDM scores (F = 0.656, *P* = 0.515, Table 3), and no significant difference between LBS and NLBS groups (F = 0.302, *P* = 0.583, Table 4) in the average RYDM scores.

**Table 3:** Test of within-subjects effects (*n* = 1101)

Source		Type III Sum of squares	df	Mean square	F	Sig.
Time	Sphericity assumed	2.129	2	1.065	14.873	0.000
	Greenhouse-Geisser	2.129	1.948	1.093	14.873	0.000
	Huynh-Feldt	2.129	1.954	1.089	14.873	0.000
	Lower-bound	2.129	1.000	2.129	14.873	0.000
Time*group	Sphericity assumed	0.094	2	0.047	0.656	0.519
	Greenhouse-Geisser	0.094	1.948	0.048	0.656	0.515
	Huynh-Feldt	0.094	1.954	0.048	0.656	0.516
	Lower-bound	0.094	1.000	0.094	0.656	0.418
Error(time)	Sphericity assumed	141.577	1978	0.072	–	–
	Greenhouse-Geisser	141.577	1927.056	0.073	–	–
	Huynh-Feldt	141.577	1932.761	0.073	–	–
	Lower-bound	141.577	989.000	0.143	–	–

* means the interaction effect of time with groups on the average RYDM scores

**Table 4:** Test of between-subjects effect (*n* = 1101)

Source	Type III Sum of squares	df	Mean square	F	Sig.
Intercept	17599.022	1	17599.022	53803.307	0.000
Group	0.099	1	0.099	0.302	0.583
Error	323.501	989	0.327	–	–

### The association between mental health and resilience

The average MHT scores in LBS were 41.54, 39.79, 38.84 in the baseline, first and second follow-up surveys, respectively, showing a continuous decline. The simple correlation coefficients between the RYDM and MHT scores were−0.227,−0.158, and−0.204 in the baseline, first and second follow-up surveys, respectively (*P* = 0.000). The difference in RYDM and MHT scores between the baseline and last follow-up surveys were calculated and the simple correlation coefficient between the difference values was−0.185 (*P* = 0.000) and the regression coefficient was−5.513 (t =−6.229, *P* = 0.000).

## Discussion

Recently, studies about the resilience of children have been gradually increasing all over the world, but the existing research focuses mostly on the theory, mechanism and the factors that influence resilience and most are cross-sectional studies [27–29]. Our study is the first prospective cohort study to examine the change in resilience of rural left-behind middle school students in the Three Gorges area in China.

The detection rate of mental health problems for middle school students in the rural Three Gorges area was 5.64 %, which was in line with one of the previous studies conducted with rural students in Chongqing, China using the MHT scale (5.5 %) [30]. In addition, relevant data shows that 10 to 30 % of Chinese middle school students have different degrees of mental health problems [31], and rates of mental disorders ranged from 8 to 57 % in global adolescents aged 12–24 years [32]. The detection rate of mental health problems in our survey was lower than both the national and international levels. Meanwhile, almost 99 % of the LBS had medium or high resilience, which was in accordance with the other studies focus on LBC [33, 34] and normal children [35]. Compared with general children, LBS in the rural Three Gorges area underwent big changes in geographic location and social environment due to immigration and being left-behind allowed these students to develop high levels of resilience, which enabled them to improve their ability, to some extent, to defend against difficulties and promote higher levels of adaptability. Thus, the mental states of LBS were relatively good.

The average RYDM scores in LBS declined gradually and the effect of time on the average scores was significant, indicating that the long-term separation from parents may have adverse effects on a child’s resilience. The longer the time of separation, the worse off the child’s resilience. Therefore, parents of LBC should try to avoid working outside of the country for long periods of time and increase the frequency of traveling back home to live with their children as much as possible, or more time should be taken to communicate with their children in order to reduce their loneliness. Meanwhile, schools may hold some activities to make their lives after class more colorful, and teachers may try to give them more love, care, support and understanding, which were proved to be effective [9, 36]. However, we found that LBS consistently scored in the middle range for resilience, indicating that they may not have been affected by the external environment or personal factors during our survey time. Meanwhile, the change in resilience might be a long-term process, which will require more time for observation. Moreover, repeated measures analysis of variance showed that there was no significant difference between LBS and NLBS in resilience over time. One possible reason might be that the influence of the overall experience of migration in the rural Three Gorges area had surpassed the effect of being left behind. Compared with the students living in other areas, the students who lived in the rural Three Gorges area underwent rapid changes in natural, social and living environments within a short time. These adverse factors might have been paradoxical, in that they might have made the students stronger and strengthened their abilities to cope with adversity. In addition, the LBS accounted for about 75 % of all middle school students in the area, and so many of their peers were in similar circumstances. Therefore, there was no significant difference in the resilience between LBS and NLBS.

The average MHT scores in LBS declined steadily, which indicated that the mental health of LBS improved overall during our survey time, while the number of students who had psychological problems and the detection rate increased gradually. One possible reason might be that the mental status of LBS matured continuously with age and their ability to cope well with adversity also strengthened over time; however, as learning stress increased and as other adverse factors continued to build up, such as long-term separation from parents, some of the students might have developed some psychological problems. Thus, targeted mental health interventions, such as group psychology counseling or face-to-face conversations, should be conducted with these students.

The negative correlation between the mental health score and resilience in the baseline, first and second follow-up investigations showed that the higher the resilience levels, the better the mental health status, and the less prone to psychological problems, which was consistent with previous studies [37, 38]. The average RYDM and MHT scores in LBS declined continuously, and a negative association between the difference values of RYDM and MHT scores between baseline and the last follow-up survey was detected, which indicated that the larger difference in resilience levels was associated with less of a difference in mental health status. That is to say, mental health status may be predicted by resilience levels, and resilience levels have a positive and protective effect on mental health status.

LBS were found to have siblings, lived in schools and were relatively poor, as compared with NLBS in the baseline survey. However, a previous study showed that these differences may not influence resilience directly [39], thus, the aforementioned socio-demographic factors might not influence our results. Overall, 1101 students were followed in our study and the rate of follow-up was 83.28 %. The changes between the baseline and the last follow-up survey showed that the students we lost were mainly senior high school students, those living in school and those who were relatively poor, especially in LBS. We assumed that students in remote, rural areas had to live in school, and that their economic statuses were relatively poor, forcing some of them to transfer to schools closer to home or, particularly for high school students, drop out of school entirely to make money. Thus, they failed to participant in our surveys. Because a previous study also showed that these differences may not influence resilience directly [39], we considered our study population to be representative of the overall population of the area.

There are several limitations in our study. Firstly, we focused on LBS in the rural Three Gorges area compared with the local NLBS. The resilience in this group may be unique, which may not be generalized to other LBS. Secondly, we observed the change of resilience in a relatively short period of time; however, the change in resilience might be a long-term process and needs more time to confirm the variation.

## Conclusions

Our study indicates that the detection rate of mental health problems for middle school students in the rural Three Gorges area is relatively low and most of the LBS in the rural Three Gorges area have medium or high resilience. The mental health status of LBS positively correlated with their resilience levels. Resilience declines gradually as time goes on, but further studies with longer time of follow-up are needed to confirm the variation of resilience.

## Abbreviations

CHKS: California Healthy Kids Survey
LBC: left-behind children
LBS: left-behind students
MHT: Mental Health Test
NLBS: non-left-behind students
RYDM: Resilience Youth Development Module
SHKPO: Safe and Healthy Kids Program Office
